# BRAF/MEK-targeted therapy in BRAF ex15 p.T599dup mutation-driven NSCLC: a case report

**DOI:** 10.1007/s00432-024-05675-9

**Published:** 2024-03-27

**Authors:** Lan Jiang, Pirong Yang, Yufeng Liu, Juan Li

**Affiliations:** Medical Oncology, Sichuan Clinical Research Center for Cancer, Sichuan Cancer Hospital and Institute, Sichuan Cancer Centre, Affiliated Cancer Hospital of University of Electronic Science and Technology of China, Chengdu, 610041 China

**Keywords:** NSCLC, BRAF, Non-V600, p.T599dup, Case report

## Abstract

BRAF mutations are found in 1–5% of non-small-cell lung cancer (NSCLC), with V600 and non-V600 accounting for approximately 50% each. It has been confirmed that targeted therapy with dabrafenib + trametinib is effective in patients with metastatic NSCLC carrying BRAF V600E mutations. Preclinical studies have shown that dabrafenib + trametinib may also have inhibitory effects on some types of non-V600E mutations, especially some class II BRAF mutations. However, the efficacy of dabrafenib + trametinib on non-V600E mutant NSCLC in clinical practice only exists in some case reports. Here, we report a case of NSCLC patient carrying BRAF ex15 p.T599dup, who showed a clinical response to the combined therapy of dabrafenib + trametinib.

## Introduction

B-RAF murine sarcoma viral oncogene homolog B1 (BRAF) is a proto-oncogene located on chromosome 7 (7q34), and is a serine/threonine protein kinase. BRAF participates in the mitogen-activated protein kinase (MAPK) cascade, which plays a crucial role in transmitting intracellular and extracellular signals, regulating cell proliferation, differentiation, and apoptosis (Patel et al. 2020). Mutations in the RAF gene phosphorylate mitogen-activated extracellular signal-regulated kinase (MEK) (downstream of the RAS–RAF–MEK–ERK pathway); the phosphorylated MEK causes sustained activation of extracellular regulated kinases (ERK), which ultimately leads to tumorigenesis. All RAF proteins have the ability to phosphorylate MEK, but BRAF is the most active (Śmiech et al. 2020).

The prevalence of BRAF mutations is 3.9% in all malignancies, with melanoma (39.7%), thyroid cancer (33.3%) and small bowel malignancies (8.9%) having the highest prevalence of mutations (Owsley et al. 2021). The incidence of BRAF mutations ranges from 1 to 5% in non-small-cell lung cancer (NSCLC), and is most common in lung adenocarcinomas, where BRAF V600E mutations account for more than 50% of all the BRAF mutation cases (O'Leary et al. 2019). Based on the BRF113928 study, the US Food and Drug Administration (FDA) approved the indication for the combination of dabrafenib (BRAF inhibitor) + trametinib (MEK inhibitor) for the treatment of advanced NSCLC patients carrying the BRAF V600E mutation in December 2017 (Odogwu et al. 2018). The incidence of non-V600E in BRAF mutations is nearly 50%, but the treatment strategy for non-V600E mutations remains limited. Currently, only some cases have reported the efficacy of RAF/MEK inhibitors in NSCLC patients with non-V600E mutations. Here, we report a case of a patient with a rare mutation in BRAF who showed a clinical response to the combination therapy of dabrafenib + trametinib.

## Case presentation

**Fig. 1:**
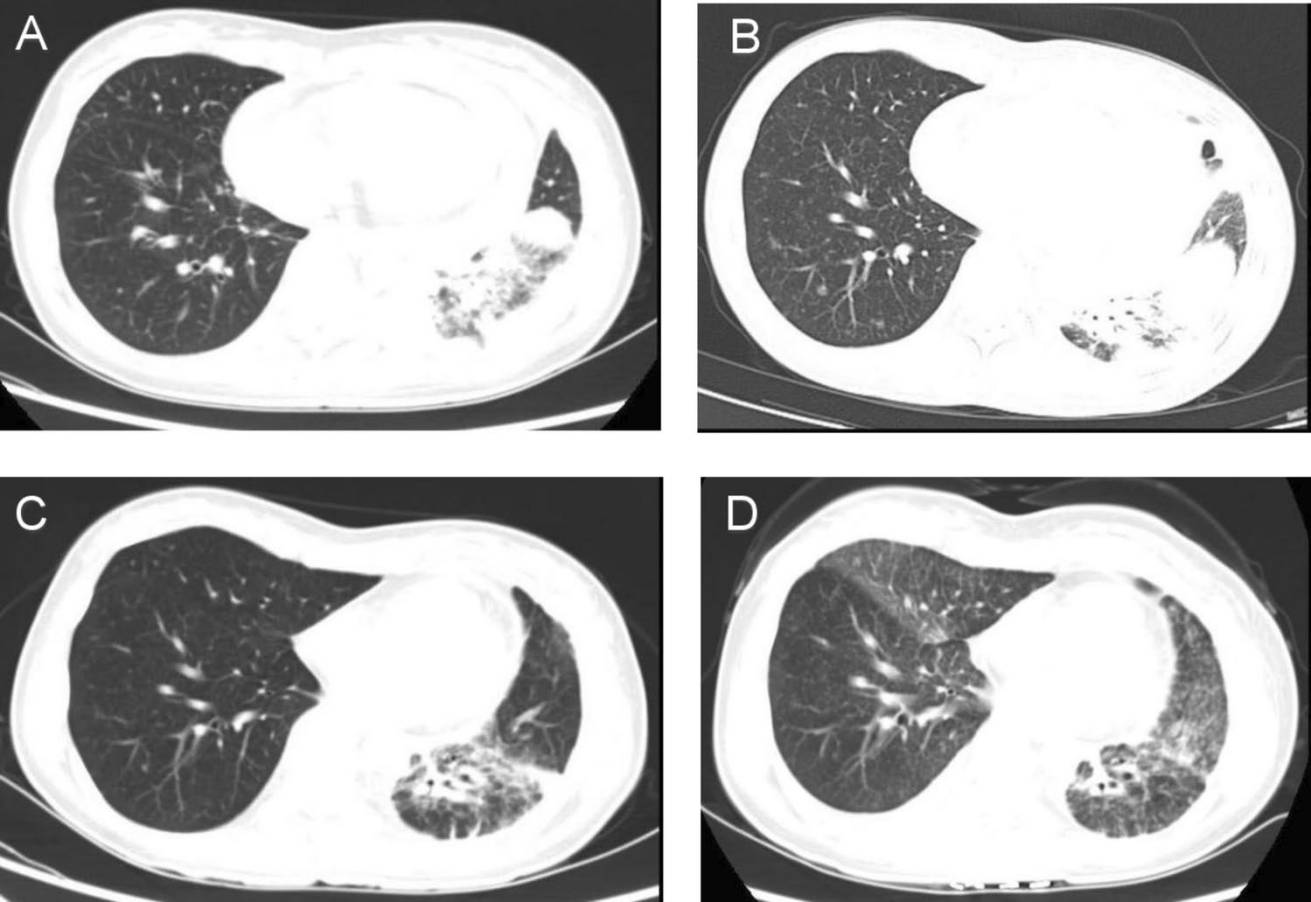
Chest CT scan results of the patient. **A** Before targeted therapy. **B** 8 months after targeted therapy: new nodules were found in both lungs. **C** 3 months after second-line treatment. **D** One year of second-line treatment. *CT* computed tomography

The patient was a 34-year-old female without a history of smoking who presented with cough and short of breath in 2021. A chest ultrasound indicated a large amount of fluid in the left pleural cavity and a small amount of fluid in the pericardium, so thoracentesis and pleural biopsy were performed. Pleural biopsy pathology suggested adenocarcinoma, immunohistochemistry showed positive TTF and negative P63. Positron emission tomography/computer tomography (PET/CT) showed a large lingual segment of the upper lobe of the left lung and a solid lesion in the lower lobe of the left lung with air bronchial signs, increased metabolism of the solid lung tissue, localized thickening of the pleura bilaterally, bilateral pleural effusion, a small amount of pericardial effusion, and an elevated SUV value (Fig. 1A). Next-generation sequencing showed BRAF exon 15 p.T599dup, TP53 exon 8 p.V272M missense mutation, BRCA2 exon 11 missense mutation andCTNNB1 exon 3 missense mutation. The patient was finally diagnosed as left lung adenocarcinoma with left pleural and pericardial metastases (cT3N0M1a, stage IVA, BRAF ex15 p.T599dup mutation positive).

**Fig. 2:**
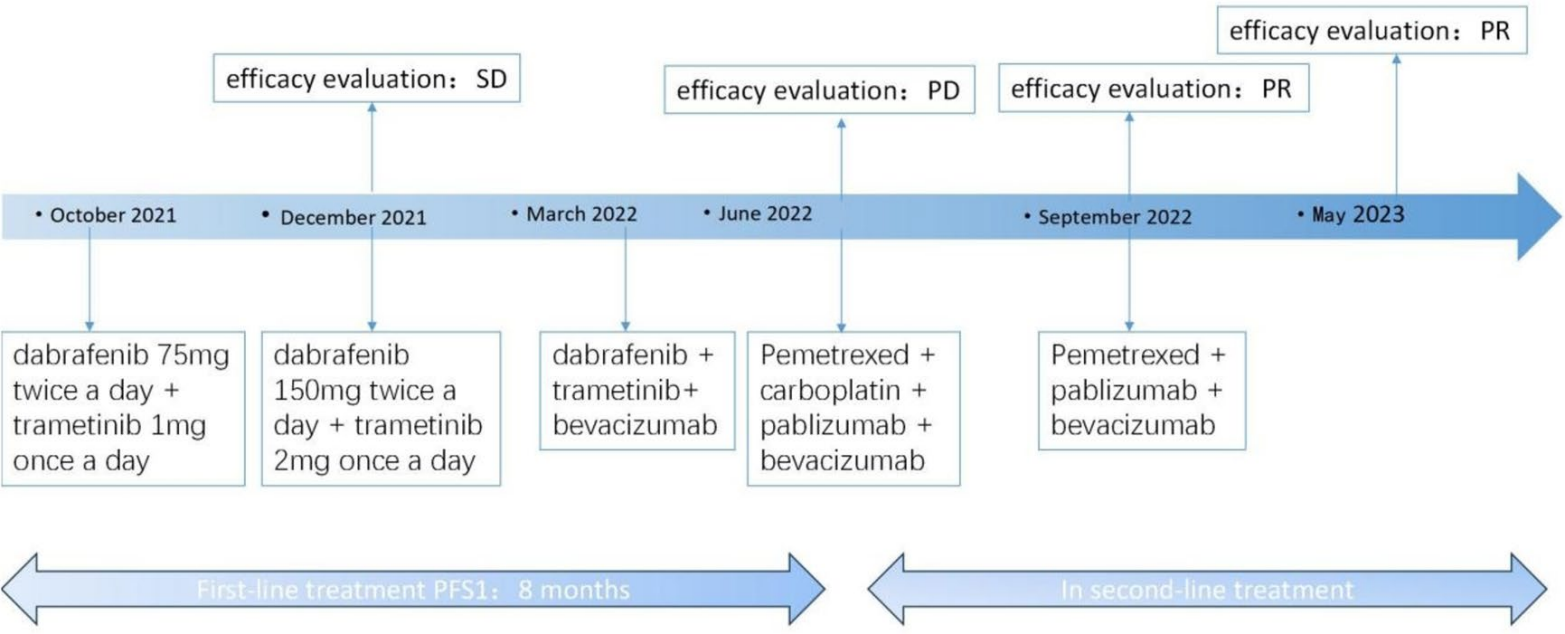
The patient’s treatment history. *SD* stable disease, *PD* progressive disease, *PR* partial response, *PFS* progression-free survival

BRAF ex15 p.T599dup belongs to class II BRAF mutation. Only one case report indicated that a patient of NSCLC with a BRAF ex15 p.T599dup mutation exhibited a durable response to the combination therapy of dabrafenib + trametinib (Turshudzhyan and Vredenburgh 2020). Considering that the patient has pericardial effusion with symptom of short of breath and low physical status (PS)score of 3, the patient decided to receive combination therapy of dabrafenib + trametinib, for the reason that time to response of targeted therapy is much faster than chemotherapy or immunotherapy. The patient was initially treated with dabrafenib and trametinib with prior patient’s consent in reduced doses (dabrafenib 75 mg twice a day and trametinib 1 mg once a day) due to a PS score of 3 (Fig. 2). The patient's adverse event during treatment was fever with a maximum temperature of 40 degrees Celsius, which returned to normal after antipyretic treatment with ibuprofen. After one month of targeted therapy, the patient’s symptoms of cough and short of breath were significantly improved, and the PS score was increased to 1. The efficacy evaluation was assessed as stable disease (SD), so the dose was adjusted to dabrafenib 150 mg twice a day and trametinib 2 mg once a day. After three months of treatment, the patient presented with symptoms of cardiac fatigue and dyspnea following physical activity, and cardiac ultrasound showed a large amount of pericardial effusion, and pericardiocentesis was performed to drain the pericardium. The morphological features of pericardial cytology suggested adenocarcinoma cells. Considering slow progression of the lung cancer with the intrapulmonary lesion did not display immediate enlargement bevacizumab was added to the targeted therapy as anti-vascular therapy. After eight months of treatment, the patient's symptoms such as cough and short of breath were worse than before, chest computed tomography (CT) scan showed new nodules in both lungs (Fig. 1B), and pericardial effusion increased than before, so the efficacy was evaluated as progression disease (PD) with 8 months of the progression-free survival 1 (PFS1). The patient’s PS score was 1 and PD-L1 expression was 5%, so pemetrexed + carboplatin + pembrolizumab + bevacizumab was chosen as patient’s second-line treatment regimen, and the best efficacy evaluation was partial response (PR) during the course of second-line treatment (Fig. 1C). After four cycles of treatment, the patient was treated with pemetrexed + pembrolizumab + bevacizumab for maintenance treatment, adverse events were 1st degree myelosuppression and gastrointestinal reactions. The patient is now on second-line treatment for 1 year and the efficacy evaluation is still in continuous PR (Fig. 1D).

## Discussion

BRAF is a component of the RAS–RAF–MEK–ERK pathway, and mutations in the BRAF gene phosphorylate MEK, which leads to sustained downstream activation and finally to tumorigenesis. BRAF mutations can be classified into the following three categories based on three aspects: whether the mutation has kinase activity, whether the kinase activity is dependent on RAS activation, and whether dimerization is required. Class I: is not dependent on upstream RAS signaling and exhibits low RAS activity due to the negative feedback of the RAS–RAF–MEK–ERK pathway. For wild-type BRAF, the formation of a BRAF dimer is required to activate the downstream pathway; whereas in class I mutations, BRAF acts as a constitutively active monomer and does not require a dimer to activate the downstream EKR pathway. The most common is the V600E mutation, in which glutamate (E) replaces valine (V) at the 600th site. Class II: Like class 1 mutations, they are also not dependent on upstream RAS signaling but require RAF dimer formation to activate the downstream EKR pathway. The location of class II mutations (activation fragment or P-loop) can block the pathway's self-inhibitory mechanism and thus maintain high kinase activity. Class II mutations include K601, L597, G464, G469, etc., and the rare mutation BRAF ex15 p.T599dup reported in this case also belongs to class II. Class III: Including G466, N581, D594, G596, etc., belonging to the kinase-impaired non-V600 mutant heterodimers with high RAS-GTP levels, which require upstream activation and transmit activation signals downstream by forming a dimer with wild-type CRAF, mutations at the DFG motif lead to impaired kinase activity (Frisone et al. 2020; Śmiech et al. 2020).

Research on targeted therapy for BRAF has focused on the V600E mutation, but class II and III BRAF mutations have also been found to be the driving mutations in lung adenocarcinoma, and the rate of non-BRAF V600E mutations can be as high as 50–80% in all BRAF mutant lung cancers (Bracht et al. 2019). Therefore, research on targeted therapy for non-BRAF V600E mutations is a breakthrough point to solve the clinical dilemma of BRAF mutation treatment. Currently, the FDA has approved three BRAF inhibitors to be used alone or in combination with other drugs in patients with BRAF V600E mutations, and the combination of dabrafenib + trametinib has been approved for patients with BRAF V600E mutations in advanced NSCLC (Poulikakos et al. 2022). In terms of the mechanism of BRAF class II and III mutations, they are all potential beneficiaries of combination therapy with RAF/MEK inhibitors, but the efficacy of combination therapy with RAF/MEK inhibitors for non-BRAF V600E mutations is currently uncertain.

To the best of our knowledge, this is the 2nd reported case of a patient with NSCLC carrying BRAF ex15 p.T599dup benefit from the combination therapy of dabrafenib + trametinib. The first patient was a 75-year-old woman diagnosed with NSCLC, a non-smoker with a history of breast cancer, reported by Alla Turshudzhyan et al. The tumor regressed significantly after 4 months of dabrafenib + trametinib treatment (Turshudzhyan and Vredenburgh 2020). Similarly, the patient in our study responded quickly from dabrafenib + trametinib treatment. After one month of treatment, the patient’s symptoms of cough and short of breath were significantly improved, and the PS score was increased from 3 to 1. One of the advantages for targeted therapy is the rapid onset of action, their time to response is much faster than chemotherapy or immunotherapy. Thus, for the patients with low PS score and need to improve symptoms quickly, targeted therapy may be a good choice. Moreover, the combination of bevacizumab and dabrafenib + trametinib can not only improve pericardial effusion, but also delaying drug resistance. After the recurrence of pericardial effusion, adding bevacizumab to dabrafenib + trametinib can make patient still benefit from targeted therapy for another 3 months.

Preclinical studies have shown that trametinib in combination with or without dabrafenib effectively inhibits cell growth in the H2087 (L597V) and H1755 (G469A) lung cancer cell lines. However, even patients with the same BRAF class II mutation may respond differently to the treatment with dabrafenib + trametinib. Patients carrying the BRAF L597R mutation were sensitive to trametinib + dabrafenib, with significant clinical benefit and sustained response for one year. Whereas patients carrying the G469V mutation were resistant to trametinib + dabrafenib, patients experienced rapid disease progression within 2 months. This may be due to the fact that patients carry a more complex gene profile (APC R1040fs∗16, CHD2 L1383∗, NFKBIA amplification, NKX2-1 amplification) (Negrao et al. 2020). Due to the diversity and functional heterogeneity of non-BRAF V600E mutations, certain locus-specific non-BRAF V600E mutations may be effective in the treatment of dabrafenib + trametinib, but only a few case reports are available at present (Reyes et al. 2019; Su et al. 2021; Turshudzhyan and Vredenburgh 2020). Both clinical and preclinical data suggest that targeted therapy with BRAF and MEK inhibitors is feasible in patients with some types of non-V600E mutations. More future studies are needed to validate the efficacy of targeted therapy in patients with this rare mutation.

## Conclusion

We report a case of a lung adenocarcinoma patient with a rare BRAF mutation who benefited from the combination therapy of dabrafenib + trametinib. We hope to draw attention to patients carrying BRAF non-V600E mutations and explore the best treatment strategy in the future.
